# Effects of small molecules on neurogenesis: Neuronal proliferation and differentiation

**DOI:** 10.1016/j.apsb.2023.10.007

**Published:** 2023-10-20

**Authors:** Michał K. Jastrzębski, Piotr Wójcik, Piotr Stępnicki, Agnieszka A. Kaczor

**Affiliations:** a Department of Synthesis and Chemical Technology of Pharmaceutical Substances with Computer Modeling Laboratory, Medical University of Lublin, Faculty of Pharmacy, Lublin PL-20093, Poland; b School of Pharmacy, University of Eastern Finland, Kuopio FI-70211, Finland

**Keywords:** Neurogenesis, Proliferation, Differentiation, Maturation, Small-molecules, Drug coctail, Alzheimer's disease, Parkinson's disease

## Abstract

Neurons are believed to be non-proliferating cells. However, neuronal stem cells are still present in certain areas of the adult brain, although their proliferation diminishes with age. Just as with other cells, their proliferation and differentiation are modulated by various mechanisms. These mechanisms are foundational to the strategies developed to induce neuronal proliferation and differentiation, with potential therapeutic applications for neurodegenerative diseases. The most common among these diseases are Parkinson's disease and Alzheimer's disease, associated with the formation of *β*-amyloid (A*β*) aggregates which cause a reduction in the number of neurons. Compounds such as LiCl, 4-aminothiazoles, Pregnenolone, ACEA, harmine, D2AAK1, methyl 3,4-dihydroxybenzoate, and shikonin may induce neuronal proliferation/differentiation through the activation of pathways: MAPK ERK, PI3K/AKT, NF*κ*B, Wnt, BDNF, and NPAS3. Moreover, combinations of these compounds can potentially transform somatic cells into neurons. This transformation process involves the activation of neuron-specific transcription factors such as NEUROD1, NGN2, ASCL1, and SOX2, which subsequently leads to the transcription of downstream genes, culminating in the transformation of somatic cells into neurons. Neurodegenerative diseases are not the only conditions where inducing neuronal proliferation could be beneficial. Consequently, the impact of pro-proliferative compounds on neurons has also been researched in mouse models of Alzheimer's disease.

## Graphical abstract

Neuronal proliferation and differentiation can be used as a treatment strategy for neurodegenerative diseases. Several low-molecular-weight chemical compounds have been described to induce neuronal cell proliferation.

**Figure:**
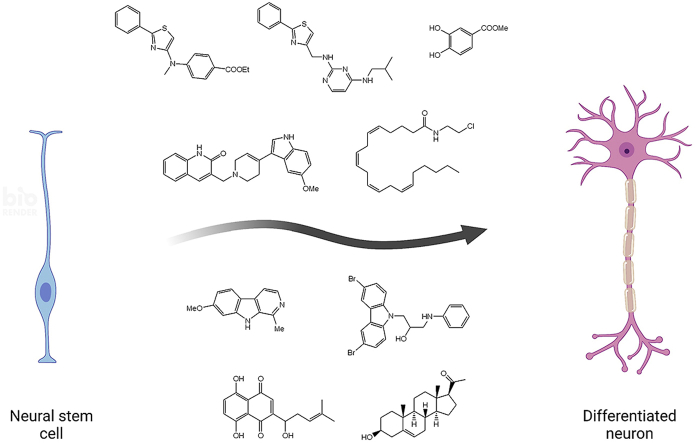

## 1 Introduction

Every cell in a multicellular organism is evolutionarily designed to play a specific role, which needs to be precisely regulated. Especially important is the regulation of the cell life cycle, including their proliferation and differentiation. Therefore, different mechanisms are evolutionarily designed for that purpose. Moreover, some cells proliferate more often, while others, after differentiation, usually do not proliferate. This situation takes place in the case of neurons, which are highly specialized cells. After being formed from stem cells during prenatal and early postnatal life, they differentiate and lose the ability to proliferate. Still, neural stem cells (NSCs) are present in the brains of adults, and under favorable conditions or if necessary, they can start to proliferate. Neural stem cells are primary progenitor cells at different developmental stages that can initiate different cell lineages, which finally lead to the formation of differentiated neurons or glial cells^1^. However, NSCs are not randomly, nor uniformly deployed in the brain. In the human brain, there are so-called neurogenic niches, where higher concentrations of NSCs are observed. As they are remnants of the embryonic germinal layer region, the microenvironment in these areas is also pro-neurogenic. In adults, two main areas of the brain are believed to be places where neurons undergo intensive proliferation: the subgranular zone (SGZ) of the hippocampus and the subventricular zone (SVZ)^2^. However, current research shows that neurogenesis also takes place in several subcortical areas, such as the hypothalamus, substantia nigra, striatum, amygdala, habenula, and cerebellum. The first phase of adult neurogenesis is the so-called precursor cell phase. At the beginning of this phase, NSCs are radial glia-like cells with triangular somas. Moreover, they express the undifferentiated neural progenitor cell (NPC) marker nestin. Then they divide asymmetrically, giving rise to transit amplifying cells, which are referred to as type II cells, and are short and wide. Type-2 cells further differentiate into type-3 cells, which are doublecortin (DCX)-positive and nestin-negative. About 3 days after the initial cell division, the amount of cells increases four times. At this stage, newly generated cells enter a post-mitotic stage characterized by the expression of post-mitotic neuronal markers: neuronal nuclei (NeuN), and calretinin. Still, massive apoptosis reduces the number of cells during the next 4 days. It is estimated that almost 80% of cells die^3^^,^^4^. Newborn neurons migrate to the destination area of the brain by following the long fibers of cells called radial glia or by chemotaxis following CCL2, CCL5, CCL12 and CC8^5^. Immature neurons produced in the SVZ can migrate along the rostral migratory stream (RMS) connecting to the olfactory bulb, where they then differentiate into mature neurons that process olfactory input, including dopaminergic cells as well as *γ*-aminobutyric acidergic (GABAergic) and glutamatergic cells^3^^,^^6^. The SGZ gives rise to granule cells of the dentate gyrus, which process information relevant to learning and memory. After that, cells undergo extensive neurite growth, during which their dendrites and axons grow. This process is promoted by GABAergic input^7^. About 12–14 days after the generation of neurons, the first dendritic synapses are formed. These processes seem to be modulated by small Rho GTPases, shifts in mitochondrial metabolism, and the disrupted in schizophrenia 1 protein (DISC1). Furthermore, surrounding cells, like astrocytes, also modulate this process^8^.

In light of the above-mentioned processes, the topic of neuronal proliferation and differentiation appears to be particularly relevant to a full understanding of the nervous system, with particular emphasis on the pathomechanisms of CNS diseases such as Alzheimer's and Parkinson's diseases. Despite the fact that the number of neuronal stem cells is rather stable, and even in old people, their number is similar to young adults, their proliferation is lower in older people, as is suggested by the lower expression of Ki67^9^, which can be caused by fluctuations in growth regulators during aging. Neuronal proliferation and differentiation are generally regulated by different growth factors like fibroblast growth factor 2 (FGF2), transforming growth factor-alpha (TGF*α*), and epidermal growth factor (EGF)^9^, ^10^, ^11^, ^12^. Among them, the production of EGF and FGF2 is different in older people compared to younger individuals. Moreover, they show high correlation with the expression of proliferation markers in stem cells. Nevertheless, insulin-like growth factor-1 (IGF-1) and glial-derived neurotrophic factor (GDNF) may also induce the proliferation of neurons^13^. On the other hand, p16. INK4a, interleukin (IL)-6, and TGF-*β*1 act in the opposite way and decrease the proliferation of neurons^13^^,^^14^.

EGF, FGF, NGF, estrogens, and G-protein receptors (GPCRs) ligands regulate neurogenesis through mitogen-activated protein kinase (MAPK) activation^15^. Activation of the Rat sarcoma virus/Rapidly Accelerated Fibrosarcoma/Mitogen-activated protein kinase kinase/extracellular signal-regulated kinases mitogen-activated protein kinase (Ras/Raf/MEK/ERK MAPK) pathway increases cell proliferation but also differentiation^16^^,^^17^. Nevertheless, despite the fact that the Ras/Raf/MEK/ERK MAPK pathway is believed to be the main regulator of neuron proliferation, other MAPKs are also involved in cell proliferation and differentiation. Also, activation of MAPK c-Jun N-terminal kinase (JNK) generally promotes apoptosis and inhibits proliferation of cells^18^, ^19^, ^20^, ^21^. Regulation of neuron proliferation may also occur in a MAPK-independent manner. For example, tumor growth factor *β* (TGF*β*) and Smad increase neuron proliferation through their own specific mechanisms^22^. On the other hand, FGF2 and TGF*α* activate another important pathway: the phosphatidylinositol 3-kinase/AKT serine/threonine kinase/mammalian target of rapamycin kinase (PI3K/AKT/mTOR) pathway, which prevents apoptosis and promotes proliferation^23^.

The aforementioned pathways are precisely regulated, but some factors can interfere with them, leading to increased or decreased neurogenesis. Among them, small molecules can be especially interesting, as it is known that some of them are able to modulate signaling pathways that are crucial in the regulation of neurogenesis, such as: SMAD, VEGF, FGF, PDGF, WNT, MAPK Erk1/2, and PI3K/Akt/mTOR signaling. Consequently, the usage of these compounds, or their mixtures, may lead to increased neurogenesis. As they are relatively easy to obtain and deliver to the brain, they may also have therapeutic properties in various diseases, in particular those linked with neuronal loss, like neurodegenerative diseases. Therefore, for a better understanding of the regulation of neurogenesis by small molecules, and their potential therapeutic properties, the exact mechanisms of action of the most promising ones are described in the following chapters.

## 2 Neurodegenerative pathomechanism of the most common diseases associated with the loss of neurons

Alzheimer's disease (AD) and Parkinson's disease (PD) are the most common diseases associated with impaired neurogenesis and, consequently, loss of neurons. Neurogenesis is affected by intrinsic cellular changes at the level of epigenetic, transcriptional or metabolic alterations^24^. Disturbances in controlling the protein quality through changes in the functioning of the autophagy-lysosome system, chaperone activity and the processing of protein aggregates contribute to neuronal death and reduced neurogenesis^25^, ^26^, ^27^. Neural stem cells (NSCs) in the hippocampus are located mainly in the subgranular zone (SGZ) of the dentate gyrus (DG) and have the ability to form new neurons. This process is impaired in AD, however, the understanding of the mechanisms underlying the reduced neurogenesis in the course of the disease is still not accurate^27^. AD is characterized by neuronal loss in the cerebral cortex and certain subcortical regions of the CNS, leading to degeneration and atrophy of the parietal and temporal lobes, cingulate gyrus, and parts of the frontal lobe^28^. The neurodegenerative pathomechanism of AD is mainly associated with the occurrence of two pathologies, namely the formation of *β*-amyloid (A*β*) aggregates and the presence of neurofibrillary tangles (NFTs)^29^. The A*β*-associated pathology results from the abnormalities in the amyloid precursor protein (APP) cleavage, leading to the formation of A*β* monomers, which in turn link into oligomeric forms of A*β*, and eventually aggregate forming A*β* fibrils and plaques^30^. In normal conditions, the APP is subjected to non-amyloidogenic proteolytic cleavage by the enzymes *α*-secretase and *λ*-secretase, resulting in the production of soluble peptides^31^. In the scenario where APP is proteolyzed by *λ*-secretase and *β*-secretase instead of *α*-secretase, insoluble peptides of amyloid *β* are produced, which, due to poor clearance, aggregate in the brain in the form of A*β* plaques^30^^,^^32^. However, the exact role of amyloid *β* in the pathogenesis of AD still remains unclear and requires further explanation as it may take up to a decade of A*β* aggregates accumulation before the first symptoms of AD occurs^29^.

**Figure 1:**
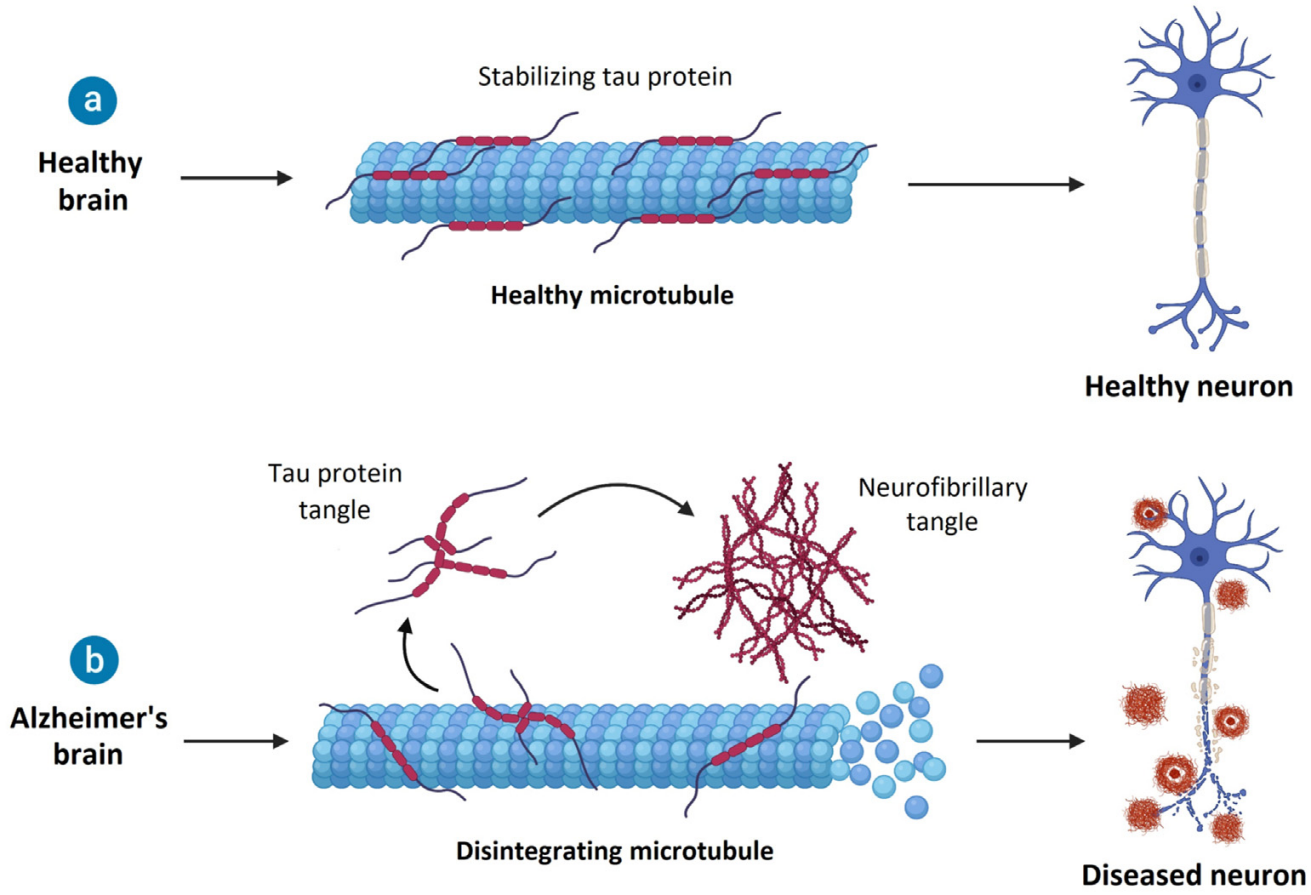
Effect of Alzheimer's disease on neurons. It has been suggested that in AD the decline in neurogenesis is exacerbated compared to normal aging. There are some earlier studies that provide conflicting evidence, suggesting that hippocampal neurogenesis in AD is increased to compensate for the neurodegenerative mechanisms^39^. However, more recent research shows that at each stage of AD there is a reduction in the number of immature neurons compared to a healthy brain. Moreover, even in the early stages, when levels of A*β* plaques and NFTs are low, a reduction in neurogenesis is observed^40^. Another study, aimed at demonstrating the correlation between cognitive performance and neurogenesis in AD, indicated that in patients with AD, the pool of hippocampal (NSCs) is reduced compared to the control group^41^.

NFTs, the second main pathology related to the development of AD, is associated with the hyperphosphorylation of the tau protein (*τ*), which is involved in the stabilization of microtubules^33^^,^^34^. The process of *τ* phosphorylation plays a crucial role in intracellular trafficking, as it enables *τ* to detach from microtubules and allows transport, while subsequent dephosphorylation leads to reattachment of *τ* to the microtubule^35^. In AD, *τ* is hyperphosphorylated, which means that the phosphorylation occurs at multiple sites, causing the *τ* to dissociate from the microtubule and leading to the breakdown of microtubule structures and disruption of many cellular processes and cell morphology^34^^,^^36^. Furthermore, the hyperphosphorylated *τ* protein aggregates and eventually form NFTs, the accumulation of which, together with the impairment of cell functioning, results in loss of neuronal function, and in consequence cell death (Fig. 1)^37^^,^^38^.

**Figure 2:**
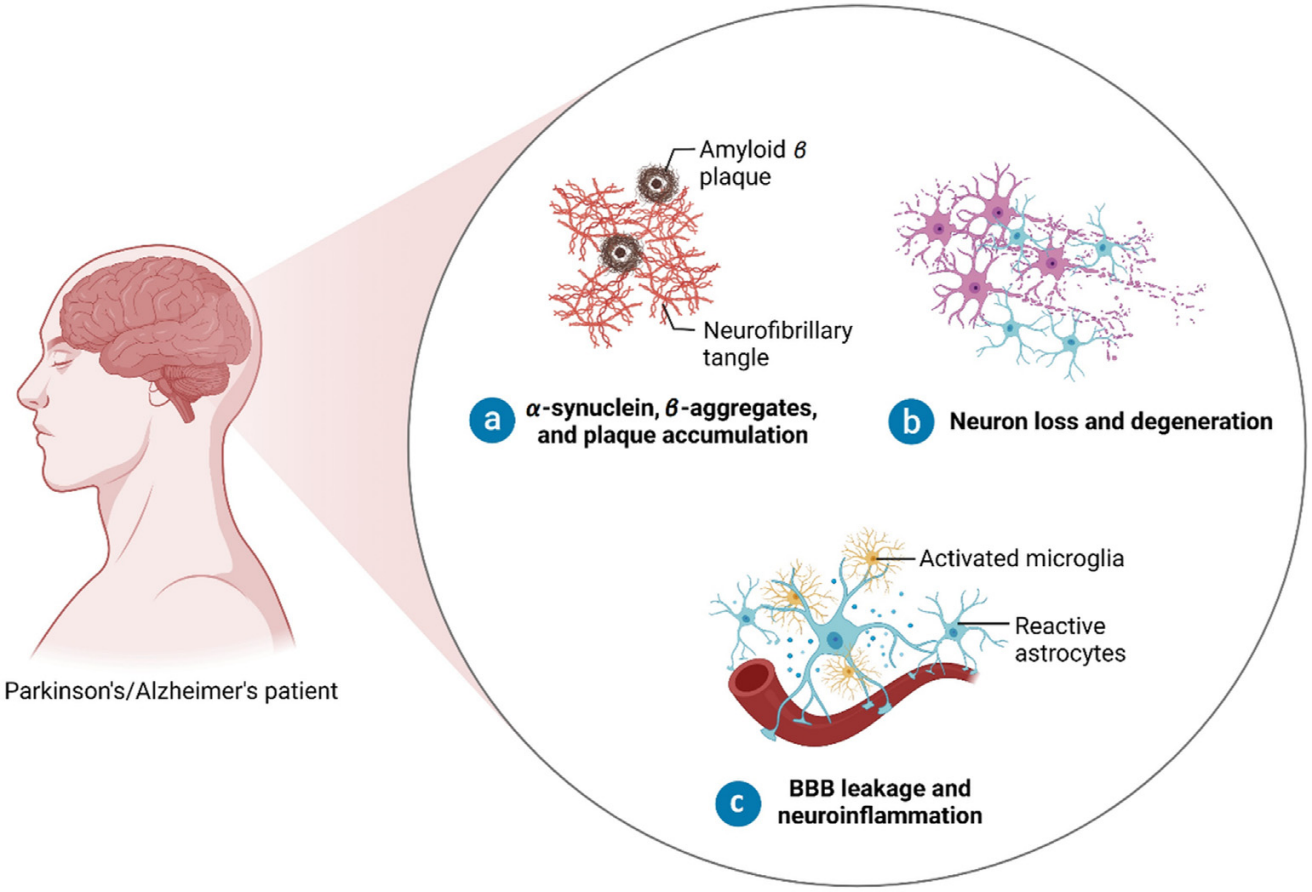
Implications of AD and PD on the central nervous system.

PD is characterized by progressive degeneration and death of motor neurons in the substantia nigra pars compacta (SNpc) within the nigrostriatal dopaminergic pathway, leading to dopamine depletion in this region^42^. In addition to the SGZ of the DG, the subventricular zone (SVZ) is a second niche for NSCs and a region in the brain where neurogenesis occurs^43^. This structure, as well as the hippocampus, are directly innervated by dopaminergic projections from the SNpc and the ventral tegmental area (VTA). Studies show that the loss of dopaminergic neurons in the SNpc in the course of PD significantly affects the proliferation of NSCs in the SVZ, leading to a decrease in neurogenesis, while pharmacological dopaminergic stimulation reverses this effect^43^^,^^44^. The main causes of neuronal loss in SNpc are impairments in protein homeostasis, leading to protein aggregation, and mitochondrial dysfunctions, associated with oxidative stress and impairments in bioenergetics^45^. Protein homeostasis is controlled by certain mechanisms, such as autophagy system, ubiquitin-protease or chaperones, which are responsible for detection, and subsequent processing or elimination of altered proteins^46^. When these surveillance mechanisms fail, native and non-native proteins tend to form aggregates, thus disturbing the homeostasis of cells (Fig. 2)^47^.

The main role in the pathogenesis of PD is attributed to *α*-Synuclein (aSyn), which is a protein disturbing the above mentioned surveillance mechanisms, leading to formation of aggregates accumulated mainly in Lewy bodies (LB) or Lewy neurites (LN). The alterations of aSyn include its phosphorylation, which increases the formation of inclusions in LB, and its misfolding, which leads to the aggregation of the protein, thus damaging the neurons in the SNpc^48^^,^^49^. One of the components of surveillance mechanisms which are reported to be affected by aSyn aggregates are heat shock protein 70 (Hsp70) and heat shock protein 40 (Hsp40), which in PD are sequestered in the LB along the aSyn, leading to loss of their function in nerve cells^50^. Furthermore, the aggregates of aSyn may inhibit RAB1A protein and the proteasome complex, thus causing impairments in the autophagy processes^45^^,^^51^. Another mechanism underlying the development of PD is associated with impairments of mitochondrial functions, in particular oxidative stress, as indicated by post-mortem studies of brains of PD patients^52^. The significant role of mitochondrial dysfunction in the pathogenesis of PD was suggested based on the fact that the exposure to 1-methyl-4-phenyl-1,2,3,4-tertahydropyridine (MPTP) results in loss of dopaminergic neurons and appearance of features characteristic of PD, which is attributed to irreversible inhibition of mitochondrial complex I^53^. The induction of PD phenotype by administration of mitochondrial complex I inhibitors, such as MPTP or rotenone, is implicated in one of the most frequently used animal models of PD in preclinical studies^54^.

Targeting mechanisms of neuronal proliferation and differentiation may lead to the development of new therapeutic strategies for the above-mentioned diseases. Therefore, it is important to develop agents that can safely and effectively promote neural cell development.

## 3 Single molecules affecting neurons

**Figure 3:**
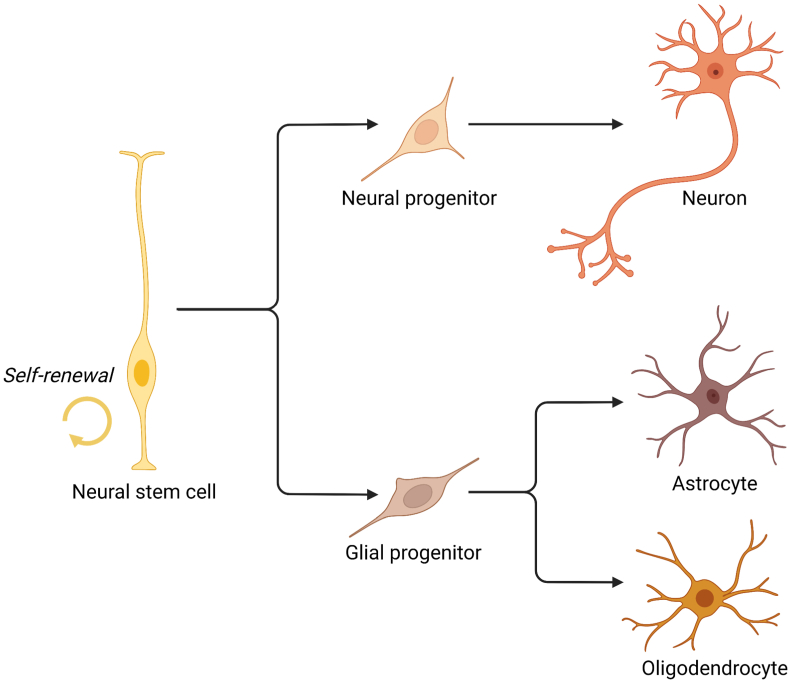
Mechanism of neural cell differentiation.

Neural stem cells (NSCs) and neural progenitor cells (NPC) have been identified in various regions of the adult brain, including the subventricular zone and the dentate cortex of the hippocampus (in the subgranular zone of the SGZ)^55^. NSCs found in both developing and adult brains are multipotent cells capable of proliferating and differentiating into primary nervous system cell types, such as neurons, astrocytes, and oligodendrocytes (Fig. 3). It is possible to functionally integrate *in vivo* obtained nervous system cells into an existing neural network^56^. However, differentiating NSCs into neuronal lineages without the action of exogenous factors, such as retinoic acid (RA) or leukemia inhibitory factor (LIF), in the absence of basic fibroblast growth factor, is challenging.

NPC-mediated neurogenesis involves at least three different processes: NPC proliferation, NPC differentiation, and survival of differentiated NPCs into neurons^57^. This mechanism provides a continuous neuronal plasticity in the brain. However, during the neurogenesis processes, many immature neurons die. Therefore, supporting the neuronal maturation process is one pathway leading to neuronal proliferation.

Each of these processes can be modulated by small molecules. There have been reports that the use of the antidepressant selective serotonin reuptake inhibitor drug, fluoxetine, is able to reverse the reduction in cell proliferation forced by the inevitable shock (IS) experiment 65. There are also reports that small molecules such as VPA can somehow enhance neuroblast cellular reprogramming in SOX2-induced neurogenesis (*in vivo* conversion of astrocytes to neurons in the injured adult spinal cord)^58^ and promote neuronal maturation^59^. Deciphering the molecular mechanisms that regulate NPCs allows better tailoring of small molecules that can either inhibit or stimulate them, while affecting all the constituent parts of neurogenesis. The Wnt signalling pathway plays a nonnegligible role in controlling neurogenesis and NSC differentiation^60^. Its activation is associated with the accumulation of *β* catenin its downstream molecule—in the cytoplasm, which is mediated also by glycogen synthase kinase 3 (GSK3), a member of receptor tyrosine kinase family. There are two subtypes of GSK3, *α* and *β*, encoded by different genes. The target of GSK3*β* is *β* catenin, whose level of phosphorylation is associated with neurodegeneration. When stem cell proliferation decreases, and cholinergic neuronal differentiation begins to dominate, the phosphorylation level of *β* catenin increases. Activation of glycogen synthase kinase 3*β* caused by *β*-catenin degradation inhibits cell proliferation, but increases cell differentiation. GSK-3*β* antagonizes the Wnt signaling pathway, playing a key role in central nervous system (CNS) development. The gene encoding the neural protein NPAS3 is another molecular target involved in adult mammalian neurogenesis, as suggested by experiments on *Npas3*^−/−^ mice, which exhibit behavioral abnormalities and loss of neurogenesis. Similarly, NF*κ*B and JAK/STAT signaling participate in regulating the proliferation and differentiation of self-renewing cells, including NCS^61^.

The search for low-molecular-weight drugs with proliferative effects on the nervous system is important for their potential therapeutic applications in neurological diseases such as Parkinson's disease, Alzheimer's disease or multiple sclerosis. By stimulating proliferative processes in nerve cells, these drugs can contribute to the regeneration of damaged nerve tissue and increase the level of neurotrophins, which can have a positive effect on the functioning of the nervous system. The search for small molecules with proliferative effects on the nervous system is also essential to understand proliferative processes in the nervous system and develop more effective methods of treatment of neurological diseases. Therefore, continued research on the search for low-molecular-weight drugs with proliferative effects on the nervous system is crucial for progress in the field of neurology and neuropharmacology.

**Figure 4:**
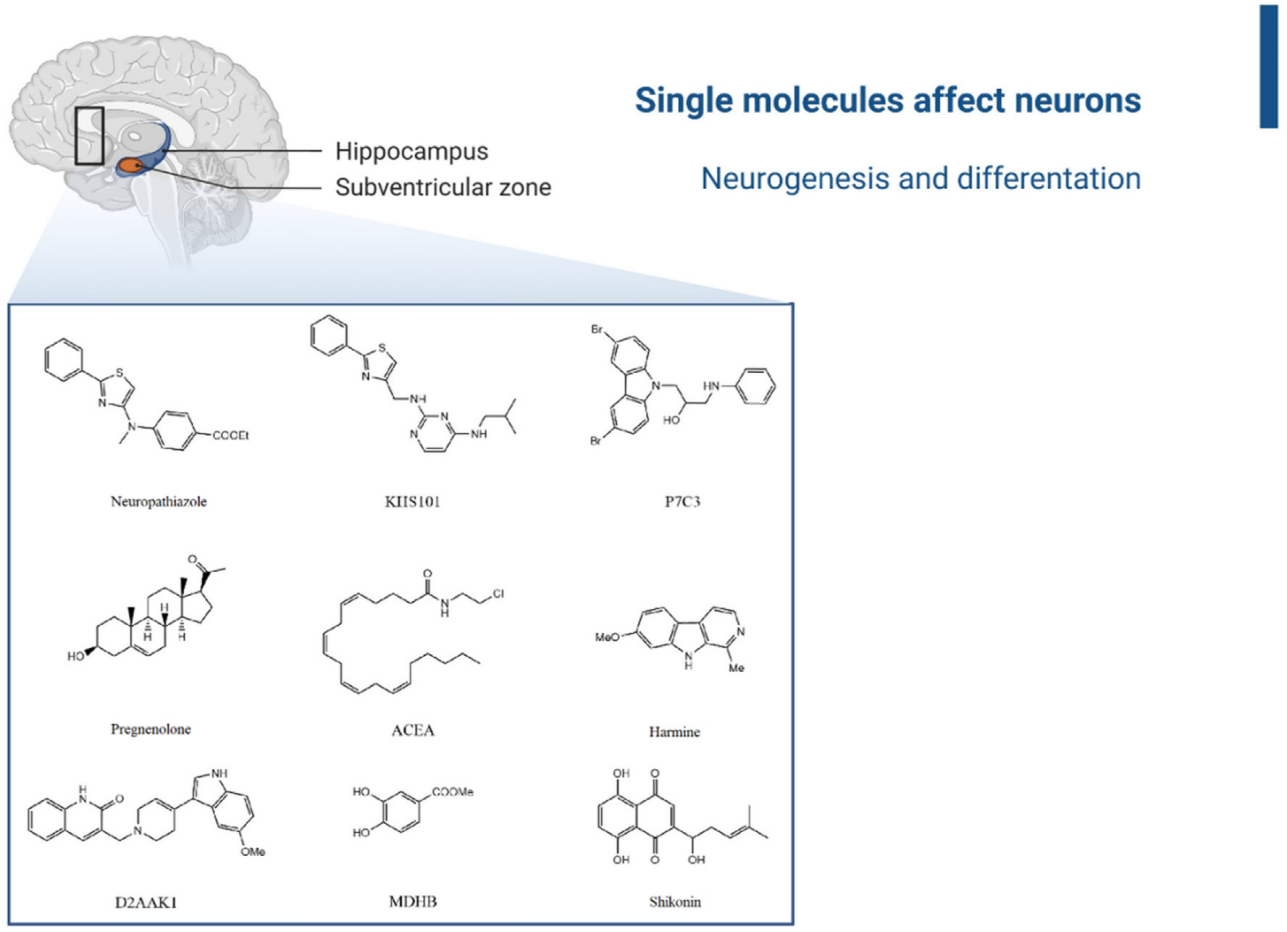
Compounds with proliferative effects: Neuropathiazole, KHS101, P7C3, Pregnenolone, ACEA, Harmine, D2AAK1, MDHB, Shikonin.

In the following section, compounds with documented proliferative and differentiating effects on neurons are presented. Their structures are listed in Fig. 4.

### 3.1 Lithium chloride

Lithium chloride is a simple inorganic compound used to treat mood disorders, in particular bipolar affective disorder. It also inhibits GSK3*β*, promoting neurogenesis during early CNS development^62^. The effects of lithium chloride on cell viability, cycle dynamics, proliferation, and differentiation of NSCs derived from rat cerebral cortex *in vitro* and *in vivo* have been reported^63^^,^^64^.

It has been shown that therapeutic lithium induces hippocampal neurogenesis and NPC proliferation in the dentate cortex^65^^,^^66^. However, transplanted NPCs have poor survival rates and mainly differentiate into glial phenotypes. Therefore, the studies demonstrating that lithium compounds enhance NPC neuronal differentiation *in vitro* and after transplantation into the dissected ventral horn of adult rats were of particular importance^63^^,^^64^. It was concluded that lithium-induced NPC neurogenesis *in vivo* is mediated by the brain-derived neurotrophic factor (BDNF) signaling pathway. ELISA, RT-PCR, and Western blotting assays revealed that lithium treatment increases the production of BDNF by NPCs in culture. Moreover, anti-BDNF antibody application decreases neurogenesis levels but does not affect NPC proliferation.

It was also demonstrated that milimolar doses of lithium chloride increase *β*-catenin expression, significantly increasing the number and size of neurospheres. NSC proliferation also significantly increases after lithium salt treatment. This study demonstrates that lithium chloride promotes NSC stem cell proliferation, survival, and maintenance *in vitro* by activating the Wnt signaling pathway.

The neuroprotective role of lithium in brain injury is probably related to the increase in transcriptional expression of the cytoprotective gene B-cell lymphoma 2 (Bcl2) and a decrease in the transcription of the proapoptotic gene Bcl-2-like protein 4 (Bax) as observed in rodent brain areas and cultured cells^67^. Increased neuronal differentiation of embryonic hippocampal NPCs likely occurs through an extracellular signal-regulated kinase and cAMP response element binding protein-dependent pathway^66^.

In contrast, the primary hypotheses for the effect of lithium chloride on signaling pathways are related to BDNF protein levels and the Wnt pathway. Lithium treatment selectively activates the BDNF IV promoter in neurons, up-regulating mRNA containing exon IV of BDNF^68^^,^^69^. The relation between lithium chloride and the Wnt signaling pathway is associated with the modulation of *β*-catenin and GSK-3*β* levels. Lithium chloride significantly increases *β*-catenin expression and effectively activates the Wnt signaling pathway, whereas GSK3*β* expression decreases in a dose-dependent manner.

In summary, the data obtained from the experiments allow to draw the conclusion that the damaged environment of the CNS lacks signals to induce neurogenesis. However, it was demonstrated that lithium administration significantly enhances the neuronal differentiation of transplanted NPCs in the removed ventral horn, both *in vitro* and *in vivo*. This finding highlights lithium's capacity to increase neuronal differentiation in NPCs. Additionally, it's important to note that BDNF plays a significant role in modifying the hostile environment of the mature CNS to promote neuronal development and plasticity. Notably, its production increases following lithium treatment, emphasizing its therapeutic potential. Overall, these results suggest that lithium could be a valuable candidate for stimulating neurogenesis and promoting neural development in CNS injuries.

### 3.2 Neuropathiazole

A class of 4-aminothiazoles has been identified to selectively induce the differentiation of multipotent neuronal stem cells in the hippocampus, with neuropathiazole showing the highest activity^70^. Treatment of progenitor cells with it significantly decreases cell proliferation, with more than 90% of cells differentiating into neuronal cells. Prolonged treatment with neuropathiazole can induce NSC cells to differentiate into mature neurons with a characteristic morphology.

Neuropathiazole can also inhibit astroglial differentiation that is induced by LIF and bone morphogenetic protein 2 (BMP2), while RA cannot^71^^,^^72^. Neuropathiazole is a more selective inducer of neuronal differentiation than RA and can competitively inhibit astrogliogenesis by LIF/BMP2/FBS in a dose-dependent manner.

The tested small-molecule neuropathiazole induces neuronal differentiation in multipotent adult hippocampal neural progenitor cells. However, there is no evidence of direct pathological relevance related to models of neurodegenerative diseases.

### 3.3 KHS101

KHS101, a small molecule that selectively induces neuronal differentiation was found to promote NPC neuronal differentiation and inhibit their proliferation^57^. The level of NPC differentiation induced by KHS101 was qualitatively similar to that induced by the known neurogenic factors RA and BDNF under conditions of adhesion and bead formation.

The effect of small molecules based on the structure of thiazoles on neurogenesis has been investigated by a targeted structure–activity relationship study (SAR). This study led to the discovery of KHS101, a 4-aminothiazole compound with enhanced activity and improved pharmacokinetic properties that targets neuronal differentiation of cultured rat hippocampal stem cells. KHS101 induced neuronal formation under conditions of neurosphere formation from both the hippocampus and subventricular zone. This increase in neuronal differentiation from progenitor cells was confirmed by increased expression of NeuroD and panneuronal marker TuJ1, as measured by RT-PCR and immunostaining, respectively.

The mechanism of action of KHS101 was investigated using an affinity-based purification method that demonstrated its physical interaction with TACC3. TACC3 can sequester various transcription factors into the cytoplasm or centrosome, preventing their binding to gene promoters^73^. Alternatively, TACC3 can modulate the proteolytic turnover of its binding partners, providing a target for proteasome degradation. The study showed that KHS101 specifically accelerates neuronal differentiation by interacting with the TACC3 protein and promotes a functional link between KHS101 and the TACC3–ARNT2 (neural transcription factor) axis. KHS101 also inhibits astrocyte formation in cultured NPCs and may override astrocyte-promoting BMP signaling. Acceleration of neurogenesis is likely to occur through negative regulation of the cell cycle combined with activation of the neuronal differentiation program from progenitor cells.

The pharmacodynamic properties of KHS101 were also investigated. Penetration of the blood–brain barrier resulted in very low systemic exposure, with a relative bioavailability of 69 % after subcutaneous administration. Extensive distribution of the test compound into the brain was observed. Animals treated with KHS101 had no signs of disease, such as lethargy, weight loss, or increased apoptosis in cells. The studies performed in this research indicate that KHS101 administration leads to a significant increase in neuronal differentiation, as evidenced by an increase in the percentage of Brdu/NeuN double-positive cells. The resulting cells exhibit normal neuronal morphology and spontaneous spiking activity, confirming the presence of functional, maturing neurons.

Previous studies on the expression of TACC3, a protein considered as a potential molecular target for the treatment of neurodegenerative diseases (for example, acting together with Hsp70 proteins)^74^, have revealed a significant connection between its function and the regulation of progenitor cell expansion and terminal differentiation during development, as well as in the development of hippocampal cells. Consequently, the binding of KHS101 to TACC3 represents a crucial interaction that can be leveraged to influence diseases pathomechanisms.

### 3.4 P7C3

To select molecules with properties that improve neurogenesis and survival of immature neurons in the dentate bend of the mouse brain, computational methods and *in silico* studies can be used. Taking into account physicochemical and pharmacological properties, the selected compounds can be studied *in vitro* and *in vivo*. Using this approach, eight compounds with proneurogenic properties were discovered in 2010, with the active compound P7C3 (aminopropylcarbazole) showing the highest potential for beneficial pharmacological properties^75^. Its formulation for oral, intravenous and i.p. administration was feasible; its half-life, bioavailability and permeation across the blood–brain barrier were also determined.

Functional variants of the chemical derivatives of compound P7C3 were then subjected to structure–activity SAR studies (37 derivatives), resulting in one compound with superior performance (P7C3A20), and several compounds with comparable proneurogenic properties—including P7C3-OMe. Animal studies (*Npas3*^−/−^ mice) were subsequently conducted, determining that the relative number of DCX^+^ neurons increased significantly with prolonged administration of P7C3, and did not affect the abundance of hippocampal astrocytes and oligodendrocytes. This indicates that the use of the test compound after the onset of neural precursor cell proliferation enhances neuronal formation in the hippocampus of adult mice. Additionally, the effect of P7C3 on apoptosis in the hippocampus of the mice studied was investigated. The compound was shown to facilitate the repair of the granular layer of the dentate cortex by overcoming genotype-specific enhancement of apoptosis. Moreover, P7C3 and the tested derivatives P7C3A20 and R-P7C3-OMe protect mitochondrial membrane permeability and integrity, which explains the enhancement of the death process of newborn neurons^76^. Finally, a calcium-induced mitochondrial dissolution assay was performed, demonstrating that the aminopropylcarbazole tested enhances hippocampal neurogenesis, alleviating age-related cognitive decline.

Comparison of compound P7C3 with two other classes of compounds with likely anti-apoptotic properties—brominated carbazole compounds, capable of inhibiting Bid-mediated release of cytochrome *c* in isolated mitochondria^77^, and the tetrahydro-*g*-carboline (an antihistamine trade-named Dimebon)^78^^,^^79^—revealed a much greater therapeutic potential of the test compound, greater proneurogenic potency, and a lower dose of efficacy.

It is well known that normal aging in rodents is associated with impaired hippocampal neurogenesis, which, in turn, is due to an increased number of apoptotic neurons in the aged rat brain^80^^,^^81^. Therefore, Fisher's elderly rat model was used to test whether the compound P7C3 could mitigate the effects of aging *in vivo*. The study assessed retention of cognitive decline, locomotor activity, and pro-neurogenic efficacy in both short-term and chronic administration tests. After sacrificing the rats, higher levels of neurons (BrdU^+^) were observed in the dentate gyrus of P7C3-exposed rats. It can be hypothesized that the proneurogenic activity of P7C3 *in vivo* can be attributed to its ability to protect mitochondrial integrity and thereby mitigate the death of newborn neurons between the time of their birth and their functional integration into the granular layer of the dentate cortex.

This example demonstrates the effectiveness of combining high-throughput screening methods (including *in silico* approaches) with *in vivo*/*in vitro* testing, to identify and optimize lead structures, allowing the design of a new class of promising therapeutic agents.

### 3.5 Pregnenolone and its derivatives

Fatty acids, cholesterol, and steroid hormones play a key role in the regulation of NSC proliferation and differentiation. For example, linoleic acid and alpha-linolenic acid can enhance the proliferation and differentiation of embryonic NSCs^82^. In the CNS, more specifically in the cerebral cortex, hippocampus, and amygdala, cholesterol is converted into pregnenolone. Additionally, its synthesis also occurs in neurons and glial cells. Pregnenolone derivatives inhibit the GABA_A_ receptor in neurons of the cerebral cortex of newborn rats^83^. Pregnenolone itself has growth-stimulating properties in neuronal cells [*via* interaction with microtubule-associated protein 2 (MAP2)], and neuroprotective properties (protection against neurotoxic kainic acid in the hippocampus). Pregnenolone metabolites, in turn, stimulate the production of new myelin sheaths, induce cell proliferation and neurogenesis in human NSCs and rat hippocampus, and a derivative, dehydroepiandrosterone (DHEA), increases the number of new neurons in the rat dentate bend^84^.

The studies on the impact of pregnenolone on NSC proliferation and differentiation demonstrated that low concentrations of pregnenolone increased NSC viability. Pregnenolone increased the number of neurospheres and cells, as evidenced by Brdu labeling. In the process of NSC differentiation under low doses of pregnenolone, cells first pass through the immature oligodendrocyte state before becoming mature MBP^+^ cells. The effect of different concentrations of the test chemical on astroglial and neuronal differentiation was also tested. At concentrations of 15 μmol/L, the number of astrocytes decreased, while at higher doses, the number of neurons examined by beta 3+ tubulin cells increased significantly^85^. The effects of the steroid pregnenolone sulfate (Preg-S) on neurogenesis, a new form of plasticity, were also investigated in young and old rats *in vivo*. Infusion of Preg-S was found to stimulate neurogenesis and expression of polysialylated forms of NCAM, PSA-NCAM, in the rat dentate bend. Preg-S facilitated the production of new neurons in the hippocampus. These effects on hippocampal plasticity are mediated by modulation of GABA_A_ receptor complex present on hippocampal neuroblasts^86^. In addition, it was found that Preg-S partially protected newborn neurons from impaired survival.

Dehydroepiandrosterone (DHEA) is another derivative that increases the number of newly generated neurons and antagonizes the inhibitory effect of corticosterone on neuronal precursor proliferation^87^^,^^88^. The mechanism of action involves pregnenolone interacting with astrocyte cell surface receptors to induce the production of various astrocyte subtypes involved in inflammation within the nervous system. Pregnenolone modulates the transcription of *Notch1* and *Pax6* genes^89^. Increased transcription of these genes is associated with increased neuronal differentiation. Inhibition of Notch1 has been reported to reduce neuronal differentiation in adult rodents, demonstrating an important role in neurogenesis, while also affecting the maintenance of hippocampal stem cells. The signalling molecule Pax has also been implicated in neuronal patterning and neurogenesis, being promoted by the Notch1 pathway^90^^,^^91^.

### 3.6 ACEA

The cannabinoid receptor 1 (CB1) is a G-protein-coupled receptor (GPCR) expressed mainly in the plasma membrane of neurons, which tightly regulates neuronal metabolism, activity, and function^92^^,^^93^. Early studies have shown that the cannabis derivative Δ9-tetrahydrocannabinol (THC) can induce neuroprotection by up-regulating the CB1 receptor and maintaining mitochondrial function^94^^,^^95^. However, the use of THC may be limited due to its side effects such as addiction, psychoactivity, tolerance, and cytotoxicity^96^. In the search for other compounds with similar effects on the CB1 receptor, arachidonyl-2-chloroethylamide (ACEA) was discovered to significantly increase mitochondrial CB1 (mtCB1) expression in neurons and hippocampal tissue^97^. *In vitro*, ACEA restored cell viability, inhibited reactive oxygen species (ROS) generation, reduced lactate dehydrogenase (LDH) release, and decreased apoptosis, improving mitochondrial function. *In vivo*, ACEA improved neurological outcomes, reduced the number of TUNEL-positive neurons, and decreased the expression of cleaved caspase 3, confirming its anti-apoptotic action. Additionally, mtCB1 activation attenuated Ca^2+^-induced neuronal mitochondrial damage^97^. However, it is worth mentioning that the benefits induced by ACEA are partially blocked by the selective, cell-impermeable haemopressin^98^, a CB1 receptor antagonist, and completely blocked by the selective, cell-penetrating CB1 receptor antagonist AM251^99^.

Influencing cannabinoid receptors may play a pivotal role in affecting the pathomechanisms of neurodegenerative diseases. These receptors regulate neurotransmitter release and synaptic strength, and they undergo activation and overexpression during disorders such as Alzheimer's disease, multiple sclerosis, amyotrophic lateral sclerosis, Parkinson's disease, and Huntington's chorea^100^. It is also known that oxidative stress and mitochondrial dysfunction are the main features of degenerative brain diseases, so affecting ROS may play an important role in treating CNS diseases^101^.

### 3.7 Harmine

*Ayahuasca* is a plant with psychotropic effects that is used in local ceremonies by Amazonian peoples. Careful study of the plant decoction has identified the active agents—harmine and harmaline, *β*-carboline alkaloids^102^. Harmine has been shown to have antidepressant and anti-anxiety effects. Preliminary studies in rodents have shown that harmine reduces depressive symptoms and restores normal levels of hippocampal neurotrophic factor BDNF^103^^,^^104^.

Studies on the effects of harmine have shown that it significantly affects human NPC proliferation, increasing the number of cells by more than 70% at an optimal concentration of 7.5 μmol/L. In addition, harmine treatment increases the specific pool of neuronal precursors associated with neurogenesis in adults by more than 60%. Notably, harmine use did not affect cell death and did not cause damage to the DNA in the cells studied. The harmine studies also show that treatment with this alkaloid increases the number of early progenitor cells expressing both GFAP and nestin. This implies that harmine enhances the proliferation of radial glial cells derived from NPCs, which can generate neurons and astrocytes^105^. To assess the behavioral effects and hippocampal BDNF levels of this compound, rats were acutely exposed to harmine. The use of this low-molecular-weight drug affected the animals’ behavior in the forced swim and open field tests, with no impact on locomotor activity. Additionally, increased BDNF protein levels were observed in the rat hippocampus.

The study found that harmine is an inhibitor of tyrosine phosphorylation-regulated kinase 1A gene with dual specificity (DYRK1A) and monoamine oxidase (MAO)^106^^,^^107^. In neurodegenerative disorders, such as Parkinson's and Alzheimer's diseases, monoamine oxidase type B (MAO-B) is believed to play a significant role in generating reactive oxygen species during the oxidation of amine substrates. It was known that treatment with MAO inhibitors (deprenyl and rasagiline) usually results in up-regulation of neurogenesis in the hippocampus, and MAO inhibition increases serotonergic neurotransmission in the adult brain. The known treatment effect of INDY, a DYRK1A inhibitor directly phosphorylates p53, attenuating the proliferation of rat and human neuronal progenitor cells^108^.

It was tested whether there is an effect of harmine analogues on hNPC proliferation related to the targets tested. The INDY inhibitor together with harmine increased hNPC proliferation, whereas pargyline, an irreversible selective MAO inhibitor, did not alter harmine-induced proliferation levels. It is therefore postulated that harmine increases the pool of neuronal progenitor cells, and DYRK1A inhibition is a possible mechanism involved in these proliferative effects. DYRK1A is an even more important target as it may affect neurogenesis not only through proliferation but also by modulating migration and neuronal differentiation^105^. The antidepressant effect of harmine may also be related to the proliferation of hypothalamic neuronal precursors and hippocampal neuronal progenitors.

The authors postulate that the action of this compound is attributed to the inhibition of monoamine reuptake. Considering the observed increase in BDNF protein levels *in vivo* and the results of behawioral studies, there is potential for harmine to be utilized as a compound affecting the mechanisms of depression and impaired neurogenesis in the patient's brain, as well as an anxiolytic drug.

### 3.8 D2AAK1

The treatment of complex diseases such as schizophrenia and other psychotic conditions requires the development of drugs that target multiple receptors, especially multiple GPCRs^109^. Computer-aided drug design techniques are the most promising method for discovering active substances with such multidirectional activities. Notably, structure- and ligand-based virtual screening (VS) techniques, as well as molecular docking supported by advanced molecular dynamics (MD) techniques and QSAR models, enable the determination of the most likely models of the dynamic processes occurring in active receptors^110^. Using these techniques, the multi-target molecule D2AAK1 [3-((4-(5-methoxy-1*H*-indol-3-yl)-3,6-dihydropyridin-1(2*H*)-yl)methyl)quinolin-2(1*H*)-one] was discovered^111^, ^112^, ^113^. Studies have demonstrated its antipsychotic, anti-anxiety, and pro-cognitive potential, explained by its multi-target nature of action, with affinity for D_1_, D_2_, D_3_, 5-HT_1A_, and 5-HT_2A_ receptors^113^^,^^114^. In a subsequent study it was also determined that D2AAK1 stimulates neuronal growth and survival and promotes neuronal integrity *in vivo*^115^.

A study conducted to assess the effects of D2AAK1 on neurons, investigating responses related to memory, locomotor activity, metabolic activity, proliferation levels, and neuronal morphology showed that D2AAK1 and its derivatives^115^ affect neuronal morphology, resulting in increased size, elongated dendrites, and denser Nissel bodies. A single injection of D2AAK1 also affects memory-related processes, and long-term pharmacotherapy promotes pro-cognitive effects. Chronic treatment does not result in changes in the hippocampus or an increase in apoptotic cells; only an increase in the number of pyramidal neurons in scrapings of treated animals was noted. The compounds increase the proliferation of mouse hippocampal HT-22 neurons and do not increase the proliferation of immature neuroblastoma cells. Interestingly, the compounds protect neuronal cells from heat by activating molecular chaperone proteins and have antioxidant properties as they reduce the concentration of both reactive oxygen species (ROS) and reactive nitrogen species (RNS) in cells. Also worth mentioning is the reduced excitotoxicity by decreasing the concentration of Ca^2+^ ions in cells.

To probe the mechanism underlying the observed effects of D2AAK1 *in silico*, the molecular similarity of the D2AAK1 structure was matched with known structures with analogous bioactivity according to the PASS program^116^. It was discovered that one of the molecular targets of D2AAK1 may be the Ca^2+^/calmodulin-dependent protein kinase I (CaMKI) delta kinase, which regulates axon elongation and growth cone motility in hippocampal and cerebellar neuronal cells. The D2AAK1 compound induces CaMKII up-regulation in SH-SY5Y and increases CaMKI levels in HT-22. These kinases are important proteins responsible for signal transduction, protein synthesis, synaptic plasticity, development, and neuronal behavior^117^.

Antagonism of D2AAK1 towards the D_2_ and 5HT_2A_ receptors is responsible for the antipsychotic effect^118^. Partial agonism towards the 5HT_1A_ and D_1_ receptors is also observed, as this multi-target action allows for the stimulation of D_1_ receptors in the prefrontal cortex, which reverses cognitive decline. Additionally, it controls the positive symptoms of schizophrenia caused by mesocortical and mesolimbic blockade of D_2_ receptors^111^. The anti-anxiety effect is likely related to the binding of D2AAK1 at the allosteric site, which alters the opening width of the 5HT_2A_ receptor input, thereby changing the kinetics of orthosteric ligand binding. High levels of neurotrophins are associated with neuronal function, survival, and development, and are also associated with the regulation of the tropomyosin-related kinase Trk signaling pathway. Under the influence of D2AAK1, Trk and TrkA receptors are upregulated for SH-SY5Y cells, suggesting selective inhibition of receptor kinase signaling^119^. The effects of D2AAK1 on long-term memory and neuronal activation have been linked to the induction of CREB phosphorylation in the BDN/CaMK/CREB signaling pathway by this compound^120^. In addition, the activation of MAPK Erk1/2 and Pi3K/Akt/mTOR signaling pathways by D2AAK1 has been confirmed^121^. Interestingly, neuroprotective effects and effects on repair mechanisms at medium concentrations of D2AAK1 are associated with Bcl-2 levels^122^. Furthermore, low levels of the transcription factor NF*κ*B are responsible for the lack of toxicity of the test compound against hippocampal cells.

Taking all of the above information into account, D2AAK1 can be used as a substance with a neuroprotective character and increasing hippocampal cell proliferation. Its cognition-promoting nature, as well as its anti-anxiety and antidepressant properties, may benefit the treatment of symptoms of neurodegenerative diseases.

### 3.9 MDHB

Methyl 3,4-dihydroxybenzoate (MDHB) is a low-molecular-weight substance with known antioxidant properties, present in extracts of classical herbs^123^. It is also known in the literature for its effects on the CNS. MDHB accelerates neurite growth of primary cortical neurons *in vitro* by inducing brain-derived neurotrophic factor expression and protects primary cortical neurons from induced apoptosis through the mitochondrial pathway^124^.

It was found that MDHB can act on the cholinergic neurotransmission system by replacing degenerated neurons with cholinergic motor neurons^125^. Neurospheres were dissociated into single NSCs, and after five days of MDHB compound treatment, cells with morphological characteristics of neurons were observed. Cells were also identified using the neuronal marker Tuj1 and the astrocyte marker GFAP. It was investigated whether MDHB induces immature neurons to mature neurons using staining with the markers MAP2 and NeuN. The experiments confirmed that MDHB enhances the differentiation of NSCs into neurons, can promote the differentiation of NSCs into mature neurons, and inhibits the differentiation of NSCs into astrocytes.

The obtained neurons were mainly the neurons of the hippocampus, as evidenced by the immune response of the neuronal markers Ctip2, Tbr1 and Prox1. Most MDHB-induced neurons were immunopositive for the cholinergic neuron marker ChAT and the motor neuron marker Isl1 and immunonegative for the marker GABAergic neurons, the marker for dopaminergic neurons TH and the marker for serotonergic neurons (5-HT). It follows that the main subtype of MDHB-induced neurons produced are cholinergic motor neurons. Induced neurons can form synapses and a neural network, as evidenced by studies on synapsin I (SYN1) and pre-additional postsynaptic early development^125^.

MDHB activates glycogen synthase kinase 3 (GSK3*β*)—associated with the regulation of NSC differentiation into adult motor neurons and the WNT pathway—and causes degradation of *β*-catenin, leading to an inability to enter the nucleus and initiate the expression of cholinergic and cell cycle-related genes^126^. In conclusion, MDHB can induce NSCs to differentiate into cholinergic neurons by regulating cell cycle-related proteins and the cholinergic signalling pathway.

As mentioned in Section 3.6, oxidative stress likely plays a significant role in the pathogenesis of neurodegenerative diseases. Consequently, antioxidant supplements have the potential to prevent or delay the progression of such diseases and reduce ROS-induced neuronal damage. Additionally, it is worth noting that oxidative DNA damage can trigger apoptosis through the endogenous apoptotic pathway in the mitochondria. Given the information presented above, MDHB, with its neurotrophic, anti-apoptotic, and anti-oxidative effects in nerve cells, holds promise for influencing the treatment of neurodegenerative diseases associated with oxidative stress. Furthermore, MDHB has demonstrated the ability to protect primary cortical neurons against induced apoptosis. Studies on MDHB have also revealed that its implementation extends the lifespan of *Caenorhabditis elegans*, providing a valuable avenue for researching aging and its influence on the pathogenesis of age-related diseases^127^.

### 3.10 Shikonin

Shikonin, a phytochemical agent from the naphthoquinone family and the main component of the root of *Lithospermum erythrorhizon*^128^ has anti-inflammatory, anti-pyretic, and anti-pain properties, and promotes wound healing^129^^,^^130^. Its pharmacological effects are mainly attributed to its ability to block NF*κ*B and STAT3^131^^,^^132^.

Under conditions of ethanol-induced neurodegeneration, NF*κ*B and STAT3 inhibitors have a stimulating effect on NSC and NCP proliferation. Shikonin stimulated the proliferation and specialization of NSCs and NCPs in both intact and chronically alcoholized mice. In fact, the use of shikonin as a stimulator of nervous tissue progenitor functions proved to be more effective than the use of appropriate synthetic selective inhibitors of signaling molecules^133^.

Blocking NF*κ*B/STAT3 with shikonin in alcoholic encephalopathy significantly increased NSC content in the subventricular zone (SVZ) and NCP. This expansion of progenitor cells resulted in increased mitotic activity and specialization of both types of progenitors. Therefore, shikonin-mediated inhibition of NF*κ*B/STAT3 promoted activation of the brain's cellular renewal system under conditions of ethanol-induced neurodegeneration. Moreover, blocking the NF*κ*B and STAT3 pathways in NSCs and NCPs stimulates their proliferation and synchronizes progenitor specialization with each other. It is known that inactivation of NF*κ*B and STAT3 leads to increased production of neurotrophins, including growth factors, by neuroglial cells^133^^,^^134^. Therefore, the coordinated activity of various compartments of the cell renewal system leads to the reconstruction of affected brain structures and the correction of locomotor activity and cognitive functions of the animals' CNS^135^.

The obtained experimental data indicate the effectiveness of neuroprotection and stimulation of neuroregeneration by inhibiting NF*κ*B/STAT3 using shikonin. Inducing the expression of the NF-*κ*B pathway, along with the synthesis of pro-inflammatory mediators, leads to the activation of kinases responsible for protein hyperphosphorylation. One such protein is a *τ*-protein, whose aggregation is associated with one of the widely accepted pathomechanisms of AD^136^. Therefore, the documented effect of shikonin may be responsible for its neuroprotective effect, attributed to its anti-inflammatory activity.

## 4 Ts65Dn mouse model associated with down syndrome (DS)

**Figure 5:**
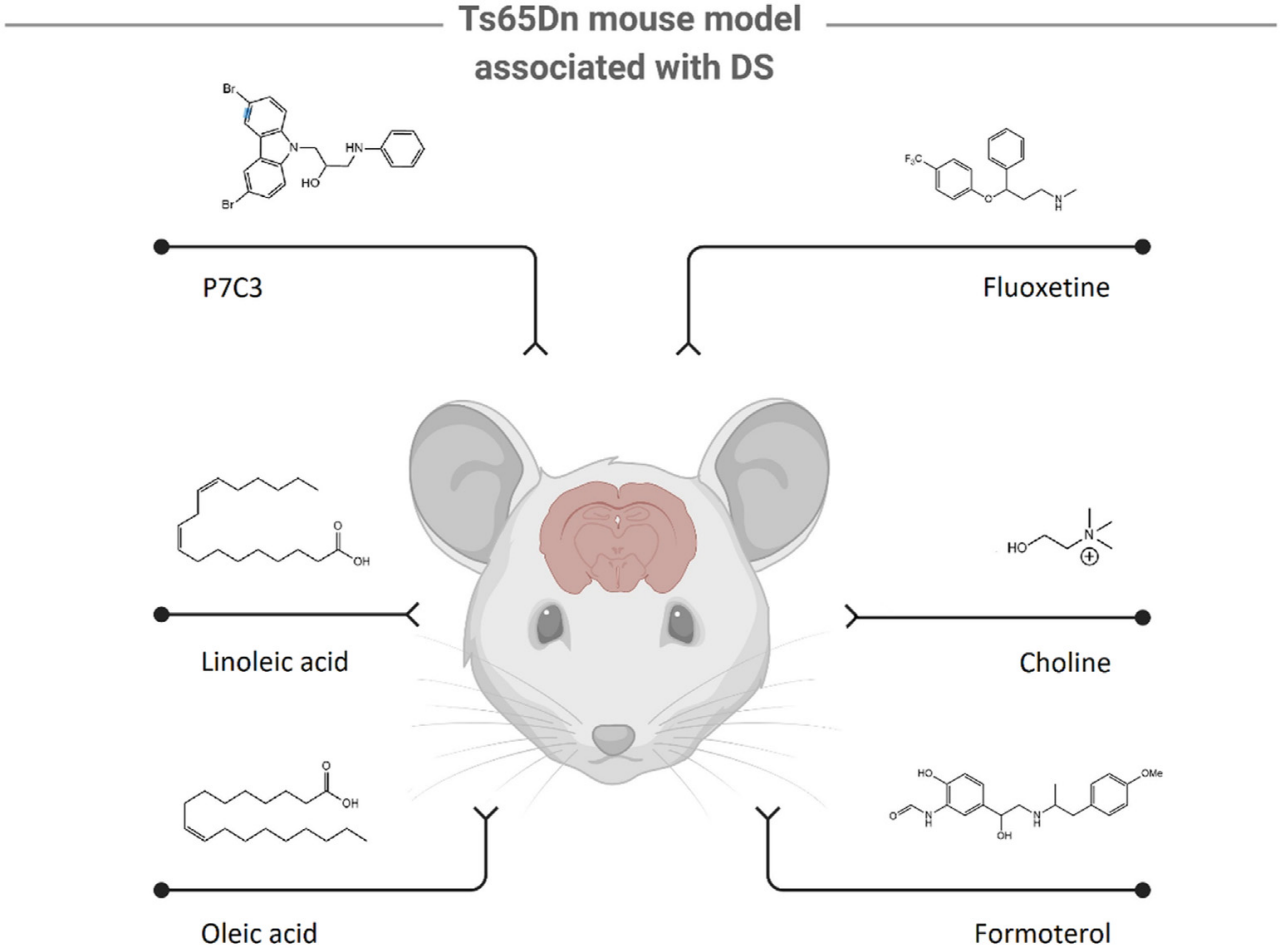
Examples of compounds used in the Ts65Dn model study.

Cognitive impairment and neuropathological features can be modeled *in vivo* using the Ts65Dn mouse model of Down syndrome. This model exhibits key features of these disorders^137^, ^138^, ^139^, including cognitive deficits, degeneration of cholinergic neurons in the basal forebrain, and a deficit in ontogenetic neurogenesis, as well as disturbed proliferation in various brain regions^140^^,^^141^. Numerous studies aim to identify compounds that improve cognitive function, increase hippocampal neurogenesis, and promote cell survival. Using the Ts65Dn model, several small molecules have been shown to have a significant impact on neurogenesis, including P7C3, LiCl, fluoxetine, choline, formoterol, and fatty acids (Fig. 5).

### 4.1 P7C3

One promising proneurogenic molecule is the aminopropylcarbazole P7C3, mentioned earlier^75^. P7C3 has the ability to increase cell survival and decrease apoptosis, thereby preventing the decline of neurogenesis and cognitive function observed in aging rats. Administration of the P7C3 compound to adult Ts65Dn mice effectively reverses the decline of neurogenesis in the adult hippocampus (measured by key markers, such as the number of Ki67, DCX^+^, BrdU^+^ cells, and the apoptotic index AC3^+^)^142^.

### 4.2 Choline

Another approach is the use of choline^143^. It is known that choline supplementation increases adult hippocampal neurogenesis in normal rats^144^. An intriguing idea was to study the use of additional choline in the diet of mother rodents in the Ts65Dn model – the addition of this low-molecular-weight active substance to the rodents' food significantly improved spatial memory, learning ability, and adult hippocampal neurogenesis of their offspring. It is postulated that the increased demand for choline during fetal development is related to its function as a precursor of membrane phospholipids and the neurotransmitter acetylcholine. Another aspect is the role of choline in DNA and histone methylation reactions, which plays an important role in gene expression regulation.^145^^,^^146^

### 4.3 Fluoxetine

The serotoninergic system plays a crucial role in neurogenesis and dendritic development. However, it is altered in the trisomic brain. The use of an SSRI drug—fluoxetine—restored neurogenesis, the number of granule cells, and dendritic morphology in the model, as well as increased cell proliferation and survival in the subgranular zone and granule cells of the hippocampal layer^147^^,^^148^. The potential mechanism by which fluoxetine restores dendritic development in trisomic mice is based on the restoration of a dysfunctional serotoninergic system. Treatment with the active compound increases serotonin availability, which indirectly reduces DSCAM protein levels and restores dendritic branching^147^^,^^149^.

### 4.4 Formoterol

Behavioral analyses have shown that formoterol, a long-acting agonist of the *β*_2_-adrenergic receptor, significantly improved cognitive function in Ts65Dn mice^150^^,^^151^. Post-mortem analyses have shown that the use of formoterol was associated with a significant improvement in synaptic density and increased complexity of newly born granule neurons in the dentate gyrus of Ts65Dn mice, as well as significant improvement in cell proliferation. Interestingly, the proliferative event was not related to the number of new neurons, as there was no increase in the number of DCX^+^ cells. Formoterol acts through Fgf2, a trophic factor synthesized by hippocampal cells, increasing the proliferative and migratory activity of glial and neuronal precursor cells. The use of *β*_2_AR agonists leads to an increase in *Fgf2* mRNA expression in the hippocampus, and treatment with formoterol increases the level of *Fgf2* mRNA, increasing the rate of hippocampal neurogenesis^152^^,^^153^.

### 4.5 Corn oil components

Linoleic acid (LA) and oleic acid (OA) are the main components of corn oil that increase cognitive performance^154^^,^^155^. It has been shown that treatment with corn oil improves hippocampal neurogenesis and hippocampus-dependent memory in a Ts65Dn DS model^156^. Evaluation of neurogenesis and dendritogenesis showed that the number of new granule cells in the dentate gyrus of the hippocampus and their dendritic pattern in treated Ts65Dn mice became similar to those in euploid mice, and their body and brain weight increased. OA supplementation has been shown to inhibit the production of A*β* peptide and amyloid plaques^157^. *In vitro* studies suggest that fatty acids act on NPCs through receptors activated by peroxisome proliferator-activated receptors *β*/*δ* and *γ*, which physically interact with the Down syndrome critical region 2 (*DSCR2*), which may be related to the mechanism of impaired neurogenesis in the patient's brain^158^.

The substances described in the aforementioned chapters 3 and 4 have been compiled into a table that summarizes their effects on neuronal cells and other activities (Table 1).

**Table 1:** Summary of substances with pro-proliferative effects on neuronal cells.

Substance	Mechanism of action	Action on neuronal cells	Other activity	*In vitro*/*in vivo* models used	Ref.
LiCl	GSK3*β* inhibition; neurogenesis is mediated by the BDNF signaling pathway; activation the Wnt signaling pathway	Enhancing the neuronal differentiation of NPCs	Treatment of mood disorders	Stem cell culture dissected out from spinal cords from wild type or transgenic Sprague–Dawley rats; embryonic NSCs collected from gestational SD rats	62, 63, 64
Neuropathiazole	Inhibition of astrogliogenesis by LIF/BMP2/FBS	Selectively inducing the differentiation of multipotent neuronal stem cells	N/a	Primary neural progenitor (HCN) cells isolated from adult rat hippocampus	70
KHS101	Inhibiting astrocyte formation in cultured npcs, overriding astrocyte-promoting BMP signaling; promotion a functional link between KHS101 and the TACC3-ARNT2	Promoting the NPC neuronal differentiation and inhibiting their proliferation	Disrupting energy metabolism in human glioblastoma cells and reducing tumor growth in mice; effects on contextual memory of morphine	Hippocampal rat NPCs	57,159
P7C3	Protecting the mitochondrial integrity (thereby mitigate the death of newborn neurons)	Increasing cell survival and decreasing apoptosis; enhancing and reversing the loss of neurogenesis	N/a	*Npas3*^−/−^ mice Fisher's elderly rat model; adult Ts65Dn mice	75
Pregnenolone derivatives	Inhibition of the GABA A receptor in neurons; interaction with MAP2; modulation of Notch1 and Pax6 gene transcription	Inducing cell proliferation, differentation and neurogenesis, modulating apoptosis	Anti-cancer activity	Appswe/PS1dE9 transgenic mice	82, 83, 84,87, 88, 89
ACEA	Significantly increasing MTCB1 expression in neurons; decreasing the expression of cleaved caspase 3	Improvement of neurological outcomes	ROS inhibition; reducing LDH	Male C57BL/6 mice	97
Harmine	DYRK1A and MAO inhibition; restoring the correct level of BDNF	Influencing proliferation and neurogenesis	Antidepressant and anti-anxiety effects;	Open-label trial conducted in an inpatient psychiatric unit; male adult Wistar rats	102, 103, 104, 105
D2AAK1	Causing upregulation of CAMKII; D_2_ and 5-HT_2A_ receptor antagonism; partial 5-HT_1A_ agonism; affecting the induction of CREB phosphorylation by the 5-HT_2A_ receptor in the BDN/camk/CREB signaling pathway; activation of MAPK Erk1/2 and PI3K/Akt/mtor signaling pathways	Neuroprotective effect and increasing the proliferation of hippocampal cells	Cognitive-promoting potential; antipsychotic, anti-anxiety and antidepressant properties; control the positive symptoms of schizophrenia	Mouse hippocampal HT-22 neurons; SH-SY5Y cells; Naive male Swiss mice	111, 112, 113, 114, 115
MDHB	Inducing BDNF expression and protecting primary cortical neurons from induced apoptosis *via* the mitochondrial pathway; activation of GSK3*β*	Promoting the differentiation of nscs into mature neurons	Anti-oxidant properties	SH-SY5Y cells	123, 124, 125
Shikonin	Blocking NF*κ*B and STAT3, affecting *τ* protein aggregation	Neuroprotective effect, attributed to its anti-inflammatory activity	Anti-pyretic, and anti-pain properties, supporting wound healing	C57B1/6 male mice; Zebrafish tumor model	129, 130, 131, 132
Choline	Influencing DNA and histone methylation reactions, choline during fetal development is associated with its function as a precursor of membrane phospholipids and the neurotransmitter ache	Increasing neurogenesis	Improvement of spatial memory and learning abilities	Ts65Dn mice	143,144
Fluoxetine	Increasing the availability of serotonin by reducing the level of the DSCAM protein	Restoring neurogenesis and dendritic morphology, increasing cell proliferation and survival	Anti-depressant and anti-cancer effect	Ts65Dn mice	147,148,160
Formoterol	Β2-adrenergic receptor agonistic activity; upregulation of FGF2 mrna in the hippocampus	Improvement of cell proliferation, synaptic density and complexity; increasing neurogenesis	Improvement of cognitive function	Adult Ts65Dn mice; Stable clonal CHO–K1 cell lines	150, 151, 152, 153
Corn oil componens	A*β* peptide production and inhibition of amyloid plaques; affecting the *β*/*δ* and *γ* receptors activated by peroxisome proliferators	Improvement of neurogenesis and dendritogenesis	Improvement of memory and cognitive performance	Ts65Dn mice	154, 155, 156, 157

## 5 Mixtures of small compounds (cocktails)

Various lineage-specific transcription factors can induce the conversion of somatic cells in mice or humans into neurons, bypassing the pluripotent state both *in vitro* and *in vivo*^161^, ^162^, ^163^. For example, NGN2 is an essential helix–loop–helix transcription factor that regulates neuronal precursor differentiation into neurons during neuron development and reprograms early postnatal astrocytes into neurons, also playing a crucial role in determining motor neuron differentiation during spinal cord development. Most studies on glia-to-neuron conversion have been conducted using ectopic, viral expression of transcription factors, which requires virus production and advanced intracranial or spinal cord injection procedures. In addition, the use of transcription factors is associated with risks such as carcinogenicity and difficulty in delivering them to the brain^164^. An alternative is to use small molecules whose biological effects are generally reversible and precisely tuned. The challenge remains in selecting a molecule or mixture of molecules (cocktails) that can convert fibroblasts or neuronal NPCs or stimulate neuron development and maturation.

A strategy based on the use of small molecules to induce cell line reprogramming would be beneficial, as it would be non-immunogenic, cost-effective, and easy to manipulate and standardize. Additionally, the application of small molecules is reversible and does not require cell permeabilization. The obvious drawbacks of such a procedure are the difficulties in conducting sequential administration, the large number of required molecules, and overall complications. Furthermore, the use of a cocktail may cause unexpected interactions and adverse effects. However, despite these problems, the introduction of small molecule drugs is considered the future of solving the causes and consequences of neurodegenerative diseases.

Astrocytes are considered an ideal starting cell type for generating new neurons due to their origin from a single progenitor cell lineage and ability to proliferate after brain injury^165^. Moreover, the feasibility of small molecule-mediated conversion of astrocytes to neurons has already been observed. Human adult astrocytes cannot spontaneously convert into doublecortin (DCX) or MAP2^+^ cells, so their changes induced by small molecules are more spectacular^164^.

The following low-molecular-weight active ingredients used in cocktails aimed at influencing neurogenesis can be included: CHIR99021, forskolin, VPA, ISX-9, IBET-151 and others. The action of individual molecules is well-described:

• ISX-9 activates the transcription of genes typical for neurons, which leads to the activation of neuronal networks^166^;
• I-BET151 represses genes associated with astrocytes and disrupts the primary transcriptional network of fibroblasts;
• VPA promotes neurogenesis and maturation of neurons by activating neuronal genes^167^;
• forskolin reduces lipid peroxidation and promotes neuronal remodeling^168^;
• CHIR99021 and inhibitors RepSox, GSK3*β*, and TGF*β* increase the efficiency of transcription factor-directed neuronal conversion^169^;
• DAPT, a *γ*-secretase inhibitor that indirectly inhibits the Notch signalling pathway, promotes neuronal differentiation^170^;
• Tzv, an inhibitor of Rho-associated kinase (ROCK), promotes cell survival and improves reprogramming efficiency^171^;
• SB431542 is an inhibitor of TGF*β*/activin receptors, which are involved in inhibiting neuronal fate and promoting glial fate^172^;
• LDN193189 is an inhibitor of BMP receptors, which are important for astroglial differentiation^173^;
• TTNPB is an agonist of RA receptors, which are crucial in neuronal patterning^174^;
• SAG and Purmo have been used to induce neuronal differentiation^175^.

The challenge remains in selecting cocktail ingredients to reduce undesirable effects, determine key components determining neurogenerative effectiveness, and analyze interactions between molecules that significantly affect the mechanism of neuronal proliferation and differentiation.

## 6 Conclusions

A great challenge in basic research as well as in treatment of neurodegenerative diseases is the fact that neuron proliferation in adults is very limited, and it decreases further during aging. Therefore, different methods have been developed to induce the proliferation and differentiation of neurons, as well as to induce the differentiation of other cells into neurons. These methods can be based on genetic engineering techniques, and on the usage of different chemicals and their mixtures. For therapeutic purposes, the most promising compounds or mixtures are those that can induce the proliferation of NSCs and their differentiation into functional neurons. For research purposes, the ability to transform widely used cells like fibroblasts into neurons seems to be more important.

The proliferation and differentiation of neural stem cells into neurons are regulated by different pathways, among which the most important ones involve MAPK ERK, Pi3K/AKT, NF*κ*B, Wnt, BDNF, and NPAS3. Therefore, the metabolism and life cycle of neural stem cells may be modulated by molecules that interact with these pathways, such as lithium chloride, 4-aminothiazoles, pregnenolone, ACEA, harmine, D2AAK1, methyl 3,4 dihydroxybenzoate, and shikonin. Activation of BDNF by lithium chloride is associated with the activation of anti-apoptotic and Wnt pathways, which not only increases the viability of cells but also promotes their differentiation into neurons. Pregnenolone also acts through BDNF activation, but in this case, additional activation of MAPK ERK, Notch1, Pax6, PI3K/Akt, and PLC is observed. Similarly, MAPK ERK and PI3K/Akt activation are also responsible for D2AAK1's impact on neurons. As these pathways regulate cell differentiation and proliferation, their activation may counteract the decreased number and activity of neurons observed in older ages.

On the other hand, combinations of compounds may have more spectacular effects, as they can transform somatic cells into fully functional neurons, mostly through indirect interactions with different genes. Every type of cell has its own individual pattern of gene expression, so modifying it may lead to the transformation of one cell into another. In the case of transforming somatic cells into neurons, the activation of various neuron-specific transcription factors like NEUROD1, NGN2, ASCL1, and SOX2 seems to be especially important. This is achieved by a cascade of upstream proteins, leading to the transcription of downstream genes and finally the transformation of somatic cells into neurons.

## Acknowledgments

This research was funded under OPUS grant from National Science Center (NCN, Poland), grant number UMO-2021/43/B/NZ7/01732 (to Agnieszka A. Kaczor). Figures were created using BioRender.

## Author contributions

Michał K. Jastrzębski drafted sections 3, 4 and 5, edited the article and created figures. Piotr Wójcik drafted sections 1 and 6 and revised the article. Piotr Stępnicki drafted section 2 and revised the article. Agnieszka A. Kaczor supervised and revised the article. All authors have read and agreed to the published version of the manuscript.

## Conflicts of interest

The authors declare no conflicts of interest.
